# Full breastfeeding protection against common enteric bacteria and viruses: results from the MAL-ED cohort study

**DOI:** 10.1093/ajcn/nqab391

**Published:** 2021-11-26

**Authors:** Benjamin J J McCormick, Stephanie A Richard, Laura E Murray-Kolb, Gagandeep Kang, Aldo A M Lima, Estomih Mduma, Margaret N Kosek, Elizabeth T Rogawski McQuade, Eric R Houpt, Pascal Bessong, Sanjaya Shrestha, Zulfiqar Bhutta, Tahmeed Ahmed, Laura E Caulfield, MAL-ED Network Investigators, Angel Mendez Acosta, Rosa Rios de Burga, Cesar Banda Chavez, Julian Torres Flores, Maribel Paredes Olotegui, Silvia Rengifo Pinedo, Mery Siguas Salas, Dixner Rengifo Trigoso, Angel Orbe Vasquez, Imran Ahmed, Didar Alam, Asad Ali, Zulfiqar A Bhutta, Shahida Qureshi, Muneera Rasheed, Sajid Soofi, Ali Turab, Aisha Yousafzai, Anita K M Zaidi, Ladaporn Bodhidatta, Geetha Ammu, Sudhir Babji, Anuradha Bose, Ajila T George, Dinesh Hariraju, M Steffi Jennifer, Sushil John, Shiny Kaki, Gagandeep Kang, Priyadarshani Karunakaran, Beena Koshy, Robin P Lazarus, Jayaprakash Muliyil, Preethi Ragasudha, Mohan Venkata Raghava, Sophy Raju, Anup Ramachandran, Rakhi Ramadas, Karthikeyan Ramanujam, Anuradha Rose, Reeba Roshan, Srujan L Sharma, Shanmuga Sundaram E, Rahul J Thomas, William K Pan, Ramya Ambikapathi, J Daniel Carreon, Viyada Doan, Christel Hoest, Stacey Knobler, Benjamin J J McCormick, Monica McGrath, Mark A Miller, Stephanie Psaki, Zeba Rasmussen, Stephanie A Richard, Jessica C Seidman, Michael Gottlieb, Dennis R Lang, Karen H Tountas, Erling Svensen, Caroline Amour, Eliwaza Bayyo, Estomih R Mduma, Regisiana Mvungi, Rosemary Nshama, John Pascal, Buliga Mujaga Swema, Ladislaus Yarrot, Carl J Mason, Tahmeed Ahmed, A M Shamsir Ahmed, Md Ashraful Alam, Rashidul Haque, Umma Haque, Md Iqbal Hossain, Munirul Islam, Mustafa Mahfuz, Dinesh Mondal, Baitun Nahar, Fahmida Tofail, Ram Krishna Chandyo, Prakash Sunder Shrestha, Rita Shrestha, Manjeswori Ulak, Aubrey Bauck, Robert E Black, Laura E Caulfield, William Checkley, Margaret N Kosek, Gwenyth O Lee, Kerry Schulze, Pablo Peñataro Yori, Laura E Murray-Kolb, A Catharine Ross, Barbara Schaefer, Suzanne Simons, Laura Pendergast, Cláudia B Abreu, Hilda Costa, Alessandra Di Moura, José Quirino Filho, Alexandre Havt, Álvaro M Leite, Aldo A M Lima, Noélia L Lima, Ila F Lima, Bruna L L Maciel, Pedro H Q S Medeiros, Milena Moraes, Francisco S Mota, Reinaldo B Oriá, Josiane Quetz, Alberto M Soares, Rosa M S Mota, Crystal L Patil, Pascal Bessong, Cloupas Mahopo, Angelina Maphula, Emanuel Nyathi, Amidou Samie, Leah Barrett, Rebecca Dillingham, Jean Gratz, Richard L Guerrant, Eric Houpt, William A Petri, James Platts-Mills, Elizabeth Rogawski, Rebecca Scharf, Elizabeth T Rogawski, Binob Shrestha, Bishnu Bahadur Rayamajhi, Sanjaya Kumar Shrestha, Tor Strand

**Affiliations:** Fogarty International Center/National Institutes of Health, Bethesda, MD, USA; Fogarty International Center/National Institutes of Health, Bethesda, MD, USA; The Pennsylvania State University, University Park, PA, USA; Christian Medical College, Vellore, India; Federal University of Ceara, Fortaleza, Brazil; Haydom Lutheran Hospital, Haydom, Manyara, Tanzania; University of Virginia, Charlottesville, VA, USA; University of Virginia, Charlottesville, VA, USA; University of Virginia, Charlottesville, VA, USA; University of Venda, Thohoyandou, South Africa; Walter Reed/AFRIMS Research Unit, Nepal, Kathmandu, Nepal; Aga Khan University, Karachi, Pakistan; icddr,b, Dhaka, Bangladesh; The Johns Hopkins Bloomberg School of Public Health, Baltimore, MD, USA; A.B. PRISMA, Iquitos, Peru; A.B. PRISMA, Iquitos, Peru; A.B. PRISMA, Iquitos, Peru; A.B. PRISMA, Iquitos, Peru; A.B. PRISMA, Iquitos, Peru; A.B. PRISMA, Iquitos, Peru; A.B. PRISMA, Iquitos, Peru; A.B. PRISMA, Iquitos, Peru; A.B. PRISMA, Iquitos, Peru; Aga Khan University, Karachi, Pakistan; Aga Khan University, Karachi, Pakistan; Aga Khan University, Karachi, Pakistan; Aga Khan University, Karachi, Pakistan; Aga Khan University, Karachi, Pakistan; Aga Khan University, Karachi, Pakistan; Aga Khan University, Karachi, Pakistan; Aga Khan University, Karachi, Pakistan; Aga Khan University, Karachi, Pakistan; Bill and Melinda Gates Foundation, Seattle, WA, USA; Armed Forces Research Institute of Medical Sciences (AFRIMS), Bangkok, Thailand; Christian Medical College, Vellore, India; Christian Medical College, Vellore, India; Christian Medical College, Vellore, India; Christian Medical College, Vellore, India; Christian Medical College, Vellore, India; Christian Medical College, Vellore, India; Christian Medical College, Vellore, India; Christian Medical College, Vellore, India; Christian Medical College, Vellore, India; Christian Medical College, Vellore, India; Christian Medical College, Vellore, India; Christian Medical College, Vellore, India; Christian Medical College, Vellore, India; Christian Medical College, Vellore, India; Christian Medical College, Vellore, India; Christian Medical College, Vellore, India; Christian Medical College, Vellore, India; Christian Medical College, Vellore, India; Christian Medical College, Vellore, India; Christian Medical College, Vellore, India; Christian Medical College, Vellore, India; Christian Medical College, Vellore, India; Christian Medical College, Vellore, India; Christian Medical College, Vellore, India; Duke University, Durham, NC, USA; Fogarty International Center/National Institutes of Health, Bethesda, MD, USA; Purdue University, Department of Nutrition Science, Lafayette, IN, USA; Fogarty International Center/National Institutes of Health, Bethesda, MD, USA; Fogarty International Center/National Institutes of Health, Bethesda, MD, USA; Fogarty International Center/National Institutes of Health, Bethesda, MD, USA; Fogarty International Center/National Institutes of Health, Bethesda, MD, USA; Fogarty International Center/National Institutes of Health, Bethesda, MD, USA; Johns Hopkins University, Baltimore, MD, USA; Fogarty International Center/National Institutes of Health, Bethesda, MD, USA; Fogarty International Center/National Institutes of Health, Bethesda, MD, USA; Leland Hunger Fellows Program, Congressional Hunger Center, Washington, DC, USA; Fogarty International Center/National Institutes of Health, Bethesda, MD, USA; Fogarty International Center/National Institutes of Health, Bethesda, MD, USA; Fogarty International Center/National Institutes of Health, Bethesda, MD, USA; Foundation for the National Institutes of Health, Bethesda, MD, USA; Foundation for the National Institutes of Health, Bethesda, MD, USA; Foundation for the National Institutes of Health, Bethesda, MD, USA; Haukeland University Hospital, Bergen, Norway; Haydom Lutheran Hospital, Haydom, Tanzania; Haydom Lutheran Hospital, Haydom, Tanzania; Haydom Lutheran Hospital, Haydom, Tanzania; Haydom Lutheran Hospital, Haydom, Tanzania; Haydom Lutheran Hospital, Haydom, Tanzania; Haydom Lutheran Hospital, Haydom, Tanzania; Haydom Lutheran Hospital, Haydom, Tanzania; Haydom Lutheran Hospital, Haydom, Tanzania; Henry M Jackson Foundation for the Advancement of Military Medicine, Bethesda, MD, USA; icddr,b, Dhaka, Bangladesh; icddr,b, Dhaka, Bangladesh; icddr,b, Dhaka, Bangladesh; icddr,b, Dhaka, Bangladesh; icddr,b, Dhaka, Bangladesh; icddr,b, Dhaka, Bangladesh; icddr,b, Dhaka, Bangladesh; icddr,b, Dhaka, Bangladesh; icddr,b, Dhaka, Bangladesh; icddr,b, Dhaka, Bangladesh; icddr,b, Dhaka, Bangladesh; Kathmandu Medical College, Kathmandu, Nepal; Institute of Medicine, Tribhuvan University, Kathmandu, Nepal; Institute of Medicine, Tribhuvan University, Kathmandu, Nepal; Institute of Medicine, Tribhuvan University, Kathmandu, Nepal; Leland Hunger Fellows Program, Congressional Hunger Center, Washington, DC, USA; Johns Hopkins University, Baltimore, MD, USA; Johns Hopkins University, Baltimore, MD, USA; Johns Hopkins University, Baltimore, MD, USA; Johns Hopkins University, Baltimore, MD, USA; University of Michigan, Department of Epidemiology, Ann Arbor, MI, USA; Johns Hopkins University, Baltimore, MD, USA; Johns Hopkins University, Baltimore, MD, USA; The Pennsylvania State University, University Park, PA, USA; The Pennsylvania State University, University Park, PA, USA; The Pennsylvania State University, University Park, PA, USA; The Pennsylvania State University, University Park, PA, USA; Temple University, Philadelphia, PA, USA; Universidade Federal do Ceara, Fortaleza, Brazil; Universidade Federal do Ceara, Fortaleza, Brazil; Universidade Federal do Ceara, Fortaleza, Brazil; Universidade Federal do Ceara, Fortaleza, Brazil; Universidade Federal do Ceara, Fortaleza, Brazil; Universidade Federal do Ceara, Fortaleza, Brazil; Universidade Federal do Ceara, Fortaleza, Brazil; Universidade Federal do Ceara, Fortaleza, Brazil; Universidade Federal do Ceara, Fortaleza, Brazil; Universidade Federal do Ceara, Fortaleza, Brazil; Universidade Federal do Ceara, Fortaleza, Brazil; Universidade Federal do Ceara, Fortaleza, Brazil; Universidade Federal do Ceara, Fortaleza, Brazil; Universidade Federal do Ceara, Fortaleza, Brazil; Universidade Federal do Ceara, Fortaleza, Brazil; Universidade Federal do Ceara, Fortaleza, Brazil; Universidade Federal do Ceara, Fortaleza, Brazil; University of Illinois at Chicago, Chicago, IL, USA; University of Venda, Thohoyandou, South Africa; University of Venda, Thohoyandou, South Africa; University of Venda, Thohoyandou, South Africa; University of Venda, Thohoyandou, South Africa; University of Venda, Thohoyandou, South Africa; University of Virginia, Charlottesville, VA, USA; University of Virginia, Charlottesville, VA, USA; University of Virginia, Charlottesville, VA, USA; University of Virginia, Charlottesville, VA, USA; University of Virginia, Charlottesville, VA, USA; University of Virginia, Charlottesville, VA, USA; University of Virginia, Charlottesville, VA, USA; University of Virginia, Charlottesville, VA, USA; University of Virginia, Charlottesville, VA, USA; University of Virginia, Charlottesville, VA, USA; Walter Reed/AFRIMS Research Unit, Kathmandu, Nepal; Walter Reed/AFRIMS Research Unit, Kathmandu, Nepal; Walter Reed/AFRIMS Research Unit, Kathmandu, Nepal; University of Bergen, Bergen, Norway

**Keywords:** breastfeeding, enteropathogens, infant feeding, enteropathy, MAL-ED

## Abstract

**Background:**

Breastfeeding is known to reduce the risk of enteropathogen infections, but protection from specific enteropathogens is not well characterized.

**Objective:**

The aim was to estimate the association between full breastfeeding (days fed breast milk exclusively or with nonnutritive liquids) and enteropathogen detection.

**Methods:**

A total of 2145 newborns were enrolled at 8 sites, of whom 1712 had breastfeeding and key enteropathogen data through 6 mo. We focused on 11 enteropathogens: adenovirus 40/41, norovirus, sapovirus, astrovirus, and rotavirus, enterotoxigenic *Escherichia coli* (ETEC), *Campylobacter* spp., and typical enteropathogenic *E. coli* as well as entero-aggregative *E. coli, Shigella* and *Cryptosporidium*. Logistic regression was used to estimate the risk of enteropathogen detection in stools and survival analysis was used to estimate the timing of first detection of an enteropathogen.

**Results:**

Infants with 10% more days of full breastfeeding within the preceding 30 d of a stool sample were less likely to have the 3 *E. coli* and *Campylobacter* spp. detected in their stool (mean odds: 0.92–0.99) but equally likely (0.99–1.02) to have the viral pathogens detected in their stool. A 10% longer period of full breastfeeding from birth was associated with later first detection of the 3 *E. coli, Campylobacter*, adenovirus, astrovirus, and rotavirus (mean HRs of 0.52–0.75). The hazards declined and point estimates were not statistically significant at 3 mo.

**Conclusions:**

In this large multicenter cohort study, full breastfeeding was associated with lower likelihood of detecting 4 important enteric pathogens in the first 6 mo of life. These results also show that full breastfeeding is related to delays in the first detection of some bacterial and viral pathogens in the stool. As several of these pathogens are risk factors for poor growth during childhood, this work underscores the importance of exclusive or full breastfeeding during the first 6 mo of life to optimize early health.

## Introduction

Given the known health advantages of breastfeeding to an infant (1), exclusive breastfeeding is recommended to 6 mo of age with continued breastfeeding to 24 mo or longer. Breast milk is a complete diet for infants aged zero to 6 mo, containing all nutrients an infant requires for healthy growth and development. However, exclusive breastfeeding often ends months earlier than recommended (1, 2) with the provision of other milks, including formula and/or solid or semi-solid food. Even when other caloric sources are not introduced, exclusive breastfeeding can be episodic with other liquids (such as water and clear nonnutritive liquids) given to the infant—a practice named predominant breastfeeding (3). Full breastfeeding includes both exclusive and predominant breastfeeding (4) and is known to confer some protection against common childhood illnesses including diarrhea (5–8).

In addition to containing nutrients, breast milk contains multiple bioactive components that can support the immunity of infants (9). For example, human-milk oligosaccharides (10, 11) inhibit pathogenic bacteria from attaching to the gut mucosal lining (12–14) and promote gut integrity (15). In addition, breast milk and individual breast-milk components alter the microbiome (16), reduce fecal bacterial diversity (17, 18), and affect gut function (19, 20).

Breastfeeding is associated with reduced diarrheal and respiratory illness, but only a few studies have examined the risk of infection with individual etiologic agents. For example, studies have shown shorter duration or reduced severity of symptoms due to pathogen-specific antibodies in breast milk (10), but not necessarily reduced likelihood of infection (21, 22). That said, 1 study of rotavirus found that severe diarrheal symptoms were delayed (to the second year of life) by full breastfeeding in a setting with high rates of exposure (23).

Using data from the Etiology, Risk Factors, and Interactions of Enteric Infections and Malnutrition and the Consequences for Child Health and Development (MAL-ED) study (24), we examine whether the duration of full breastfeeding (breast milk inclusive of consumption of nonnutritive liquids) is associated with a lower risk of pathogen detection in stool, and second, whether full breastfeeding is associated with delays in the timing of first detecting specific enteropathogens.

## Methods

### Study

The primary goal of the MAL-ED study was to describe relations among enteric infections, diet, gut function, and the growth and development of infants, and a detailed description (24) and primary results are described elsewhere (25–27). Briefly, infants were enrolled at 8 sites in low- and middle-income settings and followed to 24 mo of age. Inclusion criteria were enrollment within 17 d of birth (median: 7 d; IQR: 4–12 d), born from a singleton pregnancy to a mother at least 16 y of age, birth weight or enrollment weight >1500 g and no major morbidities, and with a family planning to stay in the community for at least 6 mo. Each site chose a target for enrollment with the aim to have data on approximately 200 children per site at 24 mo. Enrollment was staggered over 2 y. The analyses presented here were restricted to the period from birth to 6 mo, covering the recommended period of exclusive breastfeeding.

### Pathogens

Households were visited twice weekly to inquire about illness symptoms since the prior visit (28). Stools were collected when mothers reported diarrhea and also collected monthly when children were considered free of diarrhea (separated from symptoms by at least 2 d) (28). The original study protocol utilized standard techniques to identify enteropathogens in stools, but then quantitative PCR using custom-designed TaqMan Array Cards (ThermoFisher) was used to re-analyze the stool samples for the presence of 29 enteropathogens (29–31). Here, we focus on the pathogens that accounted for the majority of attributable diarrhea in the first year of life (29): adenovirus 40/41, norovirus, sapovirus, astrovirus, rotavirus, enterotoxigenic *Escherichia coli* (ETEC), *Campylobacter* spp. (pan-genus), typical enteropathogenic *E. coli* (tEPEC), *Shigella*, and *Cryptosporidium*. We additionally considered entero-aggregative *E. coli* (EAEC) as it was both frequently detected and associated with growth deficits (26). Three countries (Brazil, Peru, and South Africa) had national rotavirus vaccinations at the time of data collection and were excluded from models of rotavirus because vaccination (at ∼2 and 4 mo of age) alters the likelihood of infection and/or detection and thereby any association with breastfeeding. Following Rogawski McQuade et al. (32), pathogen presence was defined as a qPCR cycle threshold of <35. Coinfections were also identified when more than 1 pathogen was detected in a stool sample.

### Breastfeeding

An interview at enrollment asked for specific details about the timing of breastfeeding initiation, whether or not colostrum was given, and prelacteal feeding (33). During the twice-weekly surveillance visits, mothers were asked if they had breastfed the child on the previous day and whether or not other liquids or foods had been given and what foods or liquids they were. Infants who were fully breastfed were identified based on these reports. For analysis, we considered the proportion of visits that a child was fully breastfed in 2 ways. First, to determine whether full breastfeeding was associated with a lower likelihood of pathogen detection in stool, we focused on the 30-d period prior to each stool sample collection. We also continued to disaggregate time from the stool collection back to the child's enrollment in 30-d periods to evaluate period-specific associations with full breastfeeding. Second, to determine whether full breastfeeding was associated with delays in the detection of pathogens, we considered time since birth that a child was fully breastfed (exclusive of prelacteal feeding). Full breastfeeding as an exposure variable was described either as the proportion of visits between birth and when a given stool was collected or the proportion of time from birth to the age when a pathogen was first detected. In both cases, the proportion of time was multiplied by 10 to give a per 10%-time interpretation to coefficients.

### Covariates

At enrollment, and then monthly, anthropometric assessments (weight, length) were performed by trained workers using standardized protocols (34). Building on risk factors associated with specific pathogens (35–39), we controlled for child sex and weight-for-age *z* score (WAZ) at enrollment, the latter evaluated here as a continuous *z* score following the WHO growth standards (40). Some pathogen detections were also associated with aspects of lower household socioeconomic status (SES) (35, 37, 41), which was evaluated by questionnaire twice yearly. The SES metric is described in detail elsewhere (42), but briefly was defined using an index (with a range of 0, low SES, to 1, high SES) that included access to improved water and sanitation, maternal education, average monthly household income, and a range of assets or household attributes (e.g., household crowding). For the purposes of these analyses, the mean SES index across all sampling points was multiplied by 10 to examine a per 10% change in SES. In sensitivity analyses, the raw components of the metric were also examined to determine whether they had greater explanatory power than the combined construct.

### Ethics

The study was conducted in accordance with the Declaration of Helsinki. Field workers explained the study protocol and obtained written informed consent from a parent or guardian for the children enrolled in the original study. The study was approved by the following institutional review boards that correspond to each site and to collaborating institutions: Institutional Review Board for Health Sciences Research, University of Virginia, Charlottesville, VA, USA; the Committee for Ethics in Research, Universidade Federal do Ceara; National Ethical Research Committee, Health Ministry, Council of National Health in Brasília and Fortaleza—Brazil (Brazil site; BRF); Institutional Review Board, Johns Hopkins Bloomberg School of Public Health, Baltimore, MD, USA; PRISMA Ethics Committee; Health Ministry, in Loreto, Peru (Peru site; PEL); Health, Safety, and Research Ethics Committee, University of Venda; Department of Health and Social Development, Limpopo Provincial Government, in Venda, South Africa (South Africa site; SAV); Medical Research Coordinating Committee, National Institute for Medical Research; Chief Medical Officer, Ministry of Health and Social Welfare in Haydom, Tanzania (Tanzania site; TZH); Ethical Review Committee, Aga Khan University (Pakistan site in Naushahro Feroze; PKN); Ethical Review Committee, icddr,b in Dhaka—Bangladesh (Bangladesh site; BGD); Institutional Review Board, Christian Medical College, in Vellore, India, and the Health Ministry Screening Committee, Indian Council of Medical Research (India site; INV); Institutional Review Board, Institute of Medicine, Tribhuvan University; Ethical Review Board, Nepal Health Research Council; and Institutional Review Board, Walter Reed Army Institute of Research in Bhaktapur, Nepal (Nepal site; NEB).

### Statistical analysis

Site-specific descriptive characteristics of the study sample at enrollment were calculated, as well as site- and pathogen-specific distributions of child age at first detection. The proportion of visits at which full breastfeeding was reported was also calculated by site. Separate modeling approaches were used to address the following 2 questions.

First, we hypothesized that full breastfeeding would be associated with a lower likelihood of pathogen detection in stool samples. To test this, a Bayesian multivariable logistic regression model was constructed for each pathogen, with the presence or absence of the pathogen in stool as the outcome. The proportion of visits with a report of full breastfeeding during the current month was the primary exposure variable. Also included were variables for the proportion of full breastfeeding during multiple prior 30-d periods (up to 120 d). Characterized in this way, we capture influences of current full breastfeeding and a history of earlier full breastfeeding. Covariates included infant sex, WAZ at enrollment, household SES, and the count of other pathogens detected in the same stool. Infant age (months) at stool collection was included in the model. Models were further adjusted for site and individual using random effects to account for repeated measurements (an error term to account for the correlation between measurements from the same individual).

Second, we conducted a survival analysis to test the hypothesis that full breastfeeding would be associated with a delay in the time to first detection of a given pathogen. In this model, the proportion of time fully breastfed (proportion of visits from enrollment to any given stool sample) was treated as a time-varying variable (assuming a log-transformation of age) to account for potential changes with infant age in the association of full breastfeeding with first detection. Site was included as a frailty term, equivalent to a random effect in a linear regression to account for clustering in the repeated observations of each site, and covariates included sex, enrollment WAZ, SES, and the number of coincident enteropathogens.

All analyses were conducted in R 4.1.0 (R Foundation for Statistical Computing).

## Results

**FIGURE 1:**
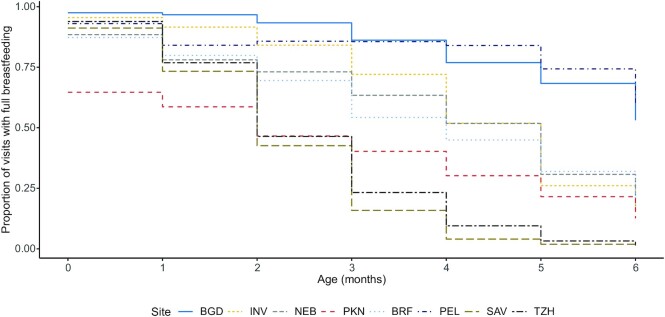
Proportion of visits recording full breastfeeding for infants in each of the first 6 mo of life by site (*n* = 1712 children). BGD, Dhaka, Bangladesh; BRF, Fortaleza, Brazil; INV, Vellore, India; NEB, Bhaktapur, Nepal; PEL, Loreto, Peru; PKN, Naushehro Feroze, Pakistan; SAV, Venda, South Africa; TZH, Haydom, Tanzania.

From the 2145 infants enrolled, 1968 were followed to at least 6 mo, of whom 1712 (87%) were included in these analyses (**Supplemental Figure 1**). Mean infant WAZ at enrollment varied across sites from −1.39 in PKN to −0.13 in TZH (**Table 1**). Although all infants in the analytic sample were initially breastfed, for most, breastfeeding was not initiated within 1 h of birth. The provision of prelacteal feeding varied greatly across sites, from 2.5% in SAV to 63% in PKN. Eighty-five percent (1313) had the expected number of twice-weekly visits through 180 d (**Supplemental Figure 2**). Overall, the proportion of visits in the first 6 mo at which full breastfeeding was reported varied from 26.0% in SAV to 94.0% in BGD (Table 1); the proportion of visits with full breastfeeding was high during the first 2 months, except in PKN, and declined over the time period (**Figure 1**).

**TABLE 1:** Selected characteristics of the analytic population^1^

	BGD	INV	NEB	PKN	BRF	PEL	SAV	TZH
*n*	210	227	226	246	164	194	236	209
Visits with full BF,^2^ median [IQR] %	94 [73, 100]	67 [48, 81]	63 [36, 83]	30 [9, 65]	56 [29, 88]	92 [77, 100]	26 [12, 43]	31 [18, 47]
Male, *n* (%)	102 (48.6)	122 (53.7)	105 (46.5)	126 (51.2)	75 (45.7)	89 (45.9)	116 (49.2)	104 (49.8)
WAZ enrollment, mean ± SD	−1.26 ± 0.94	−1.30 ± 1.04	−0.92 ± 0.97	−1.39 ± 1.05	−0.17 ± 1.05	−0.62 ± 0.91	−0.37 ± 0.94	−0.13 ± 0.94
Received colostrum, *n* (%)	206 (98.1)	204 (89.9)	219 (96.9)	204 (82.9)	161 (98.2)	188 (96.9)	228 (96.6)	194 (92.8)
BF initiated >1 h, *n* (%)	82 (39.2)	93 (41.0)	133 (58.8)	228 (93.1)	88 (53.7)	51 (26.3)	79 (38.3)	34 (16.3)
Prelacteal feeding, *n* (%)	29 (13.8)	26 (11.5)	41 (18.1)	155 (63.0)	12 (7.3)	15 (7.7)	6 (2.5)	8 (3.8)
SES,^3^ median [IQR]	0.53 [0.45, 0.63]	0.48 [0.36, 0.57]	0.70 [0.61, 0.80]	0.49 [0.35, 0.62]	0.84 [0.79, 0.90]	0.54 [0.45, 0.62]	0.79 [0.71, 0.85]	0.21 [0.14, 0.29]

1 BF, breastfeeding; BGD, Dhaka, Bangladesh; INV, Vellore, India; NEB, Bhaktapur, Nepal; PKN, Naushehro Feroze, Pakistan; BRF, Fortaleza, Brazil; PEL, Loreto, Peru; SAV, Venda, South Africa; SES, socioeconomic status; TZH, Haydom, Tanzania; WAZ, weight-for-age *z* score.

2 Over the period from enrollment to 180 days.

3 SES was measured using an in-sample index that included access to improved water and sanitation, maternal education, average monthly household income, and a range of assets (that include household crowding).

**FIGURE 2:**
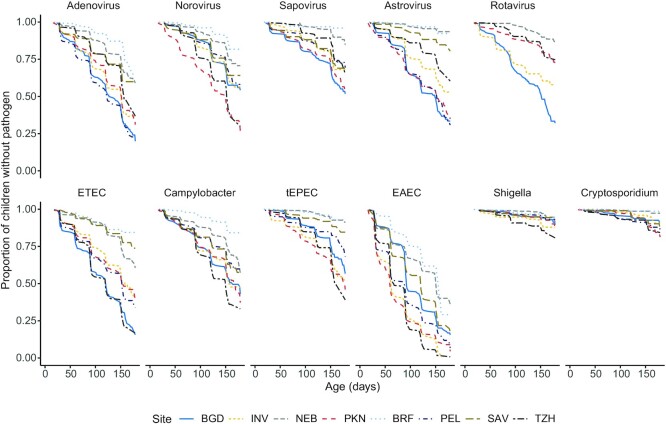
Kaplan-Meier plots of the time until selected enteropathogens were first detected at each of the 8 MAL-ED sites (*n* = 1712 children). BGD, Dhaka, Bangladesh; BRF, Fortaleza, Brazil; EAEC, entero-aggregative *Escherichia coli;* ETEC, enterotoxigenic *Escherichia coli*; INV, Vellore, India; NEB, Bhaktapur, Nepal; PEL, Loreto, Peru; PKN, Naushehro Feroze, Pakistan; SAV, Venda, South Africa; tEPEC, typical enteropathogenic *Escherichia coli*; TZH, Haydom, Tanzania.

By age 6 mo, infants in these settings experienced a mean prevalence of 14.1 d of diarrhea per child-year (varying by site from 0.6 in BRF to 50.7 in PKN). Pathogens were frequently isolated from both the monthly nondiarrheal and the diarrheal stools: the most frequently identified pathogens were EAEC and *Campylobacter* spp., with 86% and 48% of infants in the study experiencing these respective pathogens at least once in the first 6 mo of life. The timing of first pathogen detection varied across sites (**Figure 2** and **Table 2**) as did the proportion of first detections from a diarrheal as opposed to a monthly surveillance stool. The first detection of astrovirus and ETEC tended to occur around 3 mo of age and the other pathogens tended to temporally cluster around 4 mo of age (Table 2). However, the 25th percentile for first detection of many pathogens was about 60 d (median across all pathogens: 63 d), indicating that many infants were exposed and infected in the first few months of life. *Shigella* and *Cryptosporidium* had distinctly different profiles to the other pathogens analyzed here and were rarely detected in some sites during the first 6 mo of life (e.g., 2 detections of *Cryptosporidium* in BRF and just 4 in NEB). Although variable by pathogen and by site, the majority of first detections of pathogens were from a surveillance as opposed to a diarrheal stool (Table 2).

**TABLE 2:** Median [IQR] age (in days) when selected enteropathogens were first detected during the first 6 mo of life and the total number (*n*) of children with at least 1 positive test for each pathogen of which the percentage of those first detections came from diarrheal stools^1^

Pathogen	BGD (*n* = 210)	INV (*n* = 227)	NEB (*n* = 226)	PKN (*n* = 246)	BRF (*n* = 164)	PEL (*n* = 194)	SAV (*n* = 236)	TZH (*n* = 209)
Adenovirus								
Median [IQR]	92 [62, 143]	94 [62, 123]	120 [90, 151]	119 [0, 150]	121 [93, 127]	91 [60, 128]	91 [60, 122]	121 [88, 152]
*n* (% diarrhea)	146 (36.3)	128 (8.6)	77 (16.9)	146 (38.4)	25 (8)	137 (24.8)	71 (0)	103 (5.8)
Norovirus								
Median [IQR]	121 [90, 150]	120 [90, 150]	123 [96, 153]	99 [58, 138]	124 [92, 150]	122 [90, 152]	121 [89, 138]	120 [89, 151]
*n* (% diarrhea)	86 (22.1)	85 (12.9)	55 (25.5)	152 (38.8)	18 (0)	73 (26)	63 (3.2)	118 (5.1)
Sapovirus								
Median [IQR]	112 [60, 150]	121 [90, 152]	151 [100, 154]	113 [61, 152]	65 [60, 86]	116 [90, 151]	94 [59, 128]	150 [92, 156]
*n* (% diarrhea)	84 (34.5)	67 (20.9)	23 (21.7)	92 (40.2)	6 (0)	53 (26.4)	46 (2.2)	48 (12.5)
Astrovirus								
Median [IQR]	93 [76, 123]	92 [69, 124]	110 [92, 144]	89 [58, 122]	106 [83, 154]	91 [62, 143]	120 [88, 138]	120 [61, 150]
*n* (% diarrhea)	130 (26.2)	90 (23.3)	14 (21.4)	135 (54.1)	8 (0)	113 (21.2)	31 (3.2)	63 (6.3)
Rotavirus								
Median [IQR]	92 [67, 145]	90 [58, 119]	127 [110, 147]	96 [62, 150]	—	—	—	122 [88, 152]
*n* (% diarrhea)	120 (55.8)	90 (11.1)	24 (45.8)	54 (53.7)	—	—	—	44 (18.2)
ETEC								
Median [IQR]	91 [59, 123]	103 [62, 150]	148 [111, 154]	91 [58, 124]	93 [64, 121]	92 [58, 150]	94 [62, 124]	91 [61, 123]
*n* (% diarrhea)	156 (29.5)	117 (17.9)	82 (20.7)	127 (34.6)	24 (8.3)	113 (19.5)	40 (5)	157 (5.7)
*Campylobacter* spp.								
Median [IQR]	110 [70, 149]	91 [61, 137]	122 [76, 152]	100 [61, 151]	122 [93,151]	92 [60, 148]	93 [60,125]	100 [88, 127]
*n* (% diarrhea)	110 (21.8)	101 (7.9)	70 (22.9)	120 (47.5)	20 (5)	70 (35.7)	69 (2.9)	126 (4.8)
tEPEC								
Median [IQR]	124 [90, 153]	100 [60, 151]	138 [120, 151]	106 [64, 150]	122 [92, 152]	93 [60, 155]	92 [62,135]	124 [92, 154]
*n* (% diarrhea)	74 (23)	82 (12.2)	20 (35)	109 (50.5)	9 (0)	45 (33.3)	21 (14.3)	96 (4.2)
EAEC								
Median [IQR]	91 [62, 122]	61 [32, 93]	92 [59, 150]	59 [31, 89]	97 [62, 126]	61 [31, 104]	90 [60,122]	65 [58, 92]
*n* (% diarrhea)	175 (12)	213 (7)	137 (12.4)	221 (33)	75 (4)	166 (18.1)	146 (2.1)	204 (7.8)
*Shigella*/EIEC								
Median [IQR]	98 [89, 152]	94 [60, 150]	150 [115, 160]	111 [79, 151]	96 [92, 136]	98 [60, 153]	62 [60,109]	91 [70, 141]
*n* (% diarrhea)	16 (31.2)	24 (8.3)	10 (60)	20 (55)	7 (14.3)	12 (25)	11 (0)	31 (9.7)
*Cryptosporidium*								
Median [IQR]	95 [68, 127]	122 [82, 140]	107 [84, 131]	146 [121, 153]	107 [100, 114]	91 [62, 136]	123 [121,151]	93 [60, 153]
*n* (% diarrhea)	17 (5.9)	12 (25)	4 (50)	30 (60)	2 (0)	22 (22.7)	16 (0)	23 (8.7)

1 Rotavirus is not shown for the 3 sites with national vaccination. BGD, Dhaka, Bangladesh; BRF, Fortaleza, Brazil; EAEC, entero-aggregative *Escherichia coli*; EIEC, entero-invasive *Escherichia coli*; ETEC, enterotoxigenic *Escherichia coli*; INV, Vellore, India; NEB, Bhaktapur, Nepal; PEL, Loreto, Peru; PKN, Naushehro Feroze, Pakistan; SAV, Venda, South Africa; tEPEC, typical enteropathogenic *Escherichia coli*; TZH, Haydom, Tanzania.

**FIGURE 3:**
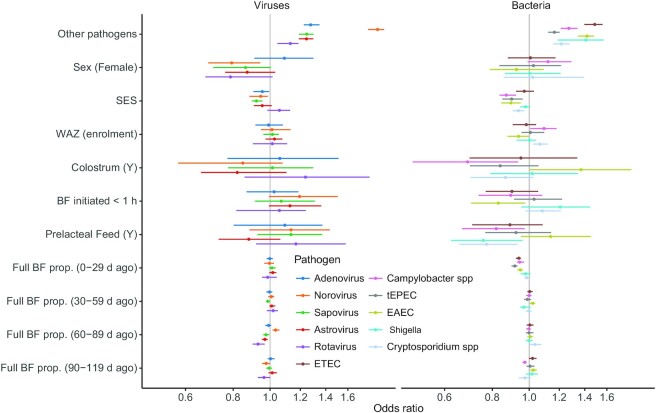
ORs (mean and 95% credibility interval) of detecting enteropathogens in stools as a function of the proportion of visits reporting full BF in 30-d periods preceding stool collection. Showing viral (left) and bacterial (right) pathogens. Logistic regression models also controlled for site as a random effect. *n* = 1712 children, except for the rotavirus model that excludes the 3 sites with routine vaccination (BRF, PEL, SAV), n = 1118. The intercept and age coefficients are not shown. BF, breastfeeding; BF initiated, whether or not breastfeeding was initiated within the first hour after birth; BRF, Fortaleza, Brazil; EAEC, entero-aggregative *Escherichia coli;* ETEC, enterotoxigenic *Escherichia coli*; Full BF prop., the proportion of visits reporting full breastfeeding from enrollment to each stool sample is considered as both a main effect and time varying term (multiplied by the log[age]); Other pathogens, a count of pathogen detected in the stool (excluding the pathogen of the response variable); PEL, Loreto, Peru; SAV, Venda, South Africa; SES, socioeconomic status index (per 10% increase, an index including water, sanitation, education, and wealth); tEPEC, typical enteropathogenic *Escherichia coli*; WAZ, weight-for-age *z* score assessed at enrollment.

The results of the logistic regression evaluating the association between full breastfeeding and the odds of detecting an enteropathogen are shown in **Figure 3**. Each 10% more visits with reported full breastfeeding in the 30 d prior to a stool sample collection was associated with significantly lower odds of detecting *Campylobacter* [0.94; 95% Credibility Interval (CrI): 0.93, 0.97], EAEC (0.95; 95% CrI: 0.93, 0.96), ETEC (0.94; 95% CrI: 0.92, 0.95), and tEPEC (0.92; 95% CrI: 0.90, 0.93) in stool. No difference in odds was found for *Shigella, Cryptosporidium*, and/or any of the 5 viral pathogens (odds of 0.99 to 1.02). Prior (more historic) full breastfeeding had inconsistent associations with the odds of pathogen detection, and this varied by site (**Supplemental Figure 3**).

**FIGURE 4:**
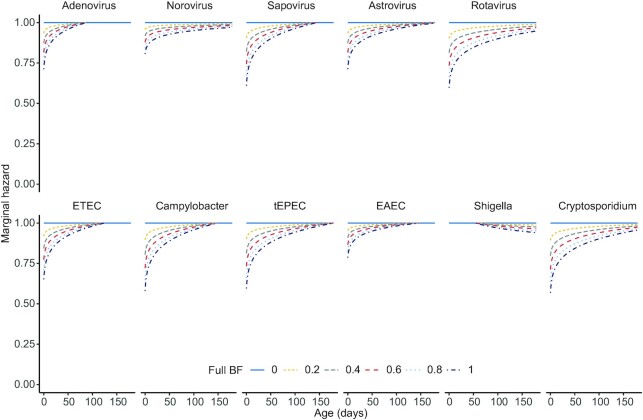
Marginal HRs for the first detection of enteropathogens as a function of the proportion of time from birth to stool sampling when the child received full breastfeeding. The survival models also adjusted for socioeconomic status (including water, sanitation, education, and wealth), weight-for-age assessed at enrollment, breastfeeding initiation, sex, and the count of coincident enteropathogens. Full breastfeeding was included as both a main effect and time-varying term (multiplied by the log[age]). Models also controlled for site using a frailty term. *n* = 1712 children except for the rotavirus model that excludes the 3 sites with routine vaccination (BRF, PEL, SAV), *n* = 1118. BF, breastfeeding; BRF, Fortaleza, Brazil; EAEC, entero-aggregative *Escherichia coli;* ETEC, enterotoxigenic *Escherichia coli*; PEL, Loreto, Peru; SAV, Venda, South Africa; tEPEC, typical enteropathogenic *Escherichia coli*.

In the survival model, full breastfeeding was associated with longer time to first infections (**Figure 4** and coefficients in **Supplemental Figure 4** and by site in **Supplemental Figure 5**). Here, the proportion of visits with full breastfeeding from enrollment to the first detection of each pathogen before 6 mo of age was evaluated, and for every 10% of time a caregiver reported full breastfeeding, the HR of first detecting most pathogens was reduced (Figure 4). The trend was for the main effect of full breastfeeding to be similarly protective of the viruses and bacteria (with the exception of *Shigella*, norovirus, and *Cryptosporidium*, 2 of which, as noted, were rare at these ages in these infants and consequently had wide CIs), with mean HRs at birth of 0.52 (95% CI: 0.37, 0.73) for *Campylobacter*, 0.54 (95% CI: 0.33, 0.87) for tEPEC, 0.60 (95% CI: 0.45, 0.80) for ETEC, 0.75 (95% CI: 0.61, 0.91) for EAEC, 0.55 (95% CI: 0.33, 0.91) for rotavirus, 0.55 (95% CI: 0.36, 0.85) for sapovirus, 0.66 (95% CI: 0.49, 0.89) for adenovirus, and 0.67 (95% CI: 0.47, 0.94) for astrovirus. The protection attributed to full breastfeeding diminished over time during the first 6 mo of life. On average, by 3 mo old, the marginal protection from full breastfeeding was predicted to have decreased substantially, ranging from 0.89 to 1.0, and was not statistically significant. The main effect and interaction with time were *P* ≤ 0.01 for all pathogens, except for adenovirus (main effect, *P* = 0.02; interaction, *P* = 0.04), norovirus (*P* = 0.22; *P* = 0.32), *Cryptosporidium* (*P* = 0.15; *P* = 0.22), and *Shigella* (*P* = 0.52; *P* = 0.48).

We included covariates in each of these models depicting variation in initial breastfeeding practices. As shown, the timing of breastfeeding initiation and whether or not colostrum was given were inconsistently associated with pathogen detections and had very wide CIs (Figure 3 and Supplemental Figure 4). A higher SES was generally protective against bacterial detection and, to a lesser extent, against viral pathogens (Figure 3 and Supplementary Figure 4), whereas female sex was protective of viral detection in some models. Coinfections in the stool samples were common; each nondiarrheal stool had a median of 1 enteropathogen detected (IQR: 0 to 2) and diarrheal samples had 2 enteropathogens (IQR: 1 to 3). Additional enteropathogen detections within the same stool were highly predictive of detection for each of the pathogens.

## Discussion

Exclusive breastfeeding of infants for the first 6 mo of life is recommended globally, both as a complete source of nutrition for infants and because of the wide-ranging health benefits, including a reduced likelihood of enteric disease. Although almost all infants in this study were breastfed, many caregivers reported full breastfeeding, meaning they reported days of exclusive breastfeeding, and on some days reported giving water and/or nonnutritive liquids. Previously, we have shown that, during an infant's first 6 mo, many caregivers begin feeding other milks and/or solids (3). Although we have shown that a pattern of days of exclusive breastfeeding of more than 50% is associated with lower risk of diarrheal illness during the period of recommended exclusive breastfeeding (8), here we focus on the more common feeding pattern of full breastfeeding and evaluate exposure to specific bacterial and viral pathogens. We present evidence that, even in settings with high rates of enteropathogen exposure, a longer duration of full breastfeeding is associated with lower odds of detection of some bacterial enteropathogens and a longer time to first infection of some bacterial and viral pathogens.

Exclusive breastfeeding may protect an infant from pathogens because of reduced oral exposure, and to the extent that water or other nonnutritive liquids are treated, the same may be true when there are intermittent days of full breastfeeding. Human milk has multiple constituents that are known to protect infants from infection and disease, including secretory IgA (sIgA) antibodies, its own microbiota, human-milk oligosaccharides, and other antimicrobial factors (e.g., lactoferrin, ɑ-lactalbumin, B-defensins), glycoproteins, and extracellular vesicles (43–45). Human milk also contains viruses that are bacteriophages (46). As recently reviewed by Nadimpalli et al. (43), there is evidence that full breastfeeding is protective of acquisition and, in some cases, of first acquisition of specific enteropathogens. Our findings add to that literature by demonstrating consistent negative associations between the extent of full breastfeeding and detection of key bacterial pathogens that are principal causes of diarrhea in these infants and worldwide. We extend those findings by showing that full breastfeeding duration is associated with later first acquisition of bacterial and viral pathogens.

The timing of infections, and hence the rate of exposure, varied by site as might be expected, and the median age at which infants started to encounter diarrheagenic pathogens was 3 mo. Many epidemiological factors contributed to this, several of which have been previously examined in this cohort for some of these pathogens and include SES and maternal education, birth weight, and the environment (35–39). Nevertheless, even accounting for intersite variability in exposures, including breastfeeding patterns, there are consistent results for some pathogens.

We found evidence that full breastfeeding (over the past 30 d) was protective against detecting 4 of the bacterial pathogens examined: *Campylobacter*, ETEC, tEPEC, and EAEC. However, current breastfeeding represents the most recent manifestation of a pattern of feeding, and older infants with a higher proportion of visits with reported full breastfeeding have a longer history of full breastfeeding. Thus, our results indicate that the longer the duration of full breastfeeding, the longer there is a lower risk of detecting pathogenic bacteria, even though, as shown (Figure 4), the protection diminishes over time during the first 6 mo of life. There is long-standing evidence that human milk can contain specific sIgA antibodies against *Campylobacter* (10) and that lactoferrin inhibits the proliferation of *E. coli* in the gut (47). In contrast, recent full breastfeeding did not show a protective effect against viral detections. One possible reason is that viral pathogens convey a stronger immune response than many of the bacterial pathogens and this reduces the likelihood of repeated infections (32).

In our second model, delay in first detection of adenovirus, sapovirus, astrovirus, and rotavirus was associated with longer duration of full breastfeeding (expressed as percentage of days from enrollment to stool collection). This is consistent with a study of rotavirus that reported a delay in the rotavirus-associated diarrhea for exclusively breastfed infants in the first 6 mo of life (23). Here, rotavirus detections were confounded by vaccination; therefore, the 3 sites with routine vaccination were excluded from the analyses. Liu et al. (48) speculated that protection against sapovirus might follow that of norovirus, for which antibodies have been detected in breast milk (49). Although the degree of full breastfeeding was not significantly associated with time to first detection of sapovirus or norovirus in these results, the mean effect was still protective, as is consistent with other research (50).

This study benefitted from consistent and rigorous quality control to produce comparable data across diverse populations. This included both the collection of breastfeeding practices through twice-weekly surveillance, as well as collection of stools during diarrhea and on nondiarrhea days. The identification of pathogens from these stools using PCR techniques allowed us to evaluate detection of specific pathogens, while considering other pathogens, and to focus on those with the most demonstrated relevance for diarrheal morbidity and growth faltering across the sites. However, a mechanistic understanding of the association between breastfeeding and enteropathogen detection was not possible because collection of human-milk samples was not part of the study protocol.

This study provides empirical evidence that full breastfeeding reduces the likelihood of acquiring 4 diarrheagenic bacterial enteropathogens in infant stools, but not viruses. Full breastfeeding also delayed the first detection of some pathogens, including both viruses and bacteria. These results underscore the importance of promoting full breastfeeding in protecting infants in settings of high rates of exposure to enteropathogens from both clinical (diarrhea) and subclinical (no diarrhea) infections, both of which have been shown to have detrimental associations with growth and development (26, 27).

## Supplementary Material

nqab391_Supplemental_File

## Data Availability

Data described in the manuscript will be made available upon request pending application and approval at http://clinepidb.org.

## Notes

The Etiology, Risk Factors and Interactions of Enteric Infections and Malnutrition and the Consequences for Child Health and Development Project (MAL-ED) is carried out as a collaborative project supported by the Bill & Melinda Gates Foundation (OPP47075), the Foundation for the NIH, and the NIH/Fogarty International Center. The findings and conclusions in this report are those of the authors and do not necessarily represent the official position of the US NIH or Department of Health and Human Services.

Supplemental Figures 1–5 are available from the “Supplementary data” link in the online posting of the article at https://academic.oup.com/ajcn/.

Abbreviations used: BGD, Dhaka, Bangladesh; BRF, Fortaleza, Brazil; CrI, credibility interval; EAEC, entero-aggregative *Escherichia coli*; EIEC, entero-invasive *Escherichia coli*; ETEC, enterotoxigenic *Escherichia coli*; MAL-ED, Etiology, Risk Factors, and Interactions of Enteric Infections and Malnutrition and the Consequences for Child Health and Development; NEB, Bhaktapur, Nepal; PKN, Naushehro Feroze, Pakistan; SAV, Venda, South Africa; SES, socioeconomic status; sIgA, secretory IgA; tEPEC, typical enteropathogenic *Escherichia coli*; TZH, Haydom, Tanzania; WAZ, weight-for-age *z* score.
