# Supplementary material for: Full breastfeeding protection against common enteric bacteria and viruses: results from the MAL-ED cohort study
Source: Am J Clin Nutr. 2021 Nov 26;115(3):759–69. doi: 10.1093/ajcn/nqab391 (PMC8895209; doi:10.1093/ajcn/nqab391)
Supplement: nqab391_Supplemental_File [file nqab391_supplemental_file.docx]

ONLINE SUPPLEMENTAL MATERIAL

**Full breastfeeding protection against common enteric bacteria and viruses: Results from the MAL-ED cohort study**

McCormick, BJJ

**Supplemental Figure 1**: Cohort profile. * One child from SAV was missing socio-economic data and was removed.

**Supplemental Figure 2**: Proportion of twice-weekly visits met.

**Supplemental Figure 3:** The odds of detecting pathogens in stools as a function of the proportion of full breastfeeding (BF) in each 30-day period preceding the stool collection. Showing the coefficients for the lagged full BF terms by site (colored dots) and overall (black triangles). Models were adjusted for site and age, SES, coincidence enteropathogens and child sex. Sites: BGD, Dhaka, Bangladesh; INV, Vellore, India; NEB, Bhaktapur, Nepal; BRF, Fortaleza, Brazil; PEL, Loreto, Peru; SAV, Venda, South Africa; TZH, Haydom, Tanzania. The rotavirus model excludes the three sites with vaccine (BRF, PEL, SAV).

**Supplemental Figure 4:** Hazard ratios of risk factors derived from survival models for time to first detection of specific pathogens. Showing viral (left) and bacterial (right) pathogens. SES, socio-economic index (per 10% increase); WAZ, weight-for-age assessed at enrolment; full BF proportion, the proportion of visits reporting full breastfeeding from enrolment to each stool sample is considered as both a main effect and time varying term (multiplied by the log(age)); BF initiated, whether or not breastfeeding was initiated within the first hour after birth. Models also controlled for site as a random effect. The rotavirus model excludes the three sites with vaccine (BRF, PEL, SAV). The intercept is not shown.

**Supplemental Figure 5:** Hazard ratios of risk factors derived from survival models for the most diarrheagenic childhood enteropathogens. SES, socio-economic index (per 10% increase); WAZ, weight-for-age assessed at enrollment; full BF proportion, the proportion of time from birth to each stool sample when the child was fully breastfed as a main effect and, where applicable, as a time varying term (multiplied by the log(age)). Models also controlled for site as a random effect. Sites: BGD, Dhaka, Bangladesh; INV, Vellore, India; NEB, Bhaktapur, Nepal; BRF, Fortaleza, Brazil; PEL, Loreto, Peru; SAV, Venda, South Africa; TZH, Haydom, Tanzania. The rotavirus model excludes the three sites with vaccine (BRF, PEL, SAV).
